# Per- and Polyfluoroalkyl Substances Exposure and Ischemic Heart Disease: Emerging Evidence from the Literature

**DOI:** 10.3390/antiox15060718

**Published:** 2026-06-05

**Authors:** Francesca Gorini, Alessandro Tonacci, Mariangela Palazzo, Elisa Bustaffa, Fabrizio Minichilli, Andrea Borghini

**Affiliations:** Institute of Clinical Physiology, National Research Council, 56124 Pisa, Italy; alessandro.tonacci@cnr.it (A.T.); mariangelapalazzo@cnr.it (M.P.); elisa.bustaffa@cnr.it (E.B.); fabrizio.minichilli@cnr.it (F.M.)

**Keywords:** PFAS, ischemic heart disease, coronary heart disease, atherosclerosis, epigenetics, telomere, mitochondrial DNA copy number, single-nucleotide polymorphisms, oxidative stress, artificial intelligence

## Abstract

Ischemic heart disease (IHD) is a chronic and progressive condition characterized by reduced blood flow, mainly due to atherosclerosis. It is currently the leading cause of mortality among cardiovascular diseases. In recent years, per- and polyfluoroalkyl substances (PFAS), a group of ubiquitous and highly persistent environmental contaminants, have emerged as potential risk factors for IHD. PFAS are well-established endocrine disruptors and have been associated with hypercholesterolemia, hypertriglyceridemia, and insulin resistance. Despite the limited number of epidemiological studies and inconsistent findings from occupational settings, accumulating evidence suggests that elevated exposure to certain PFAS compounds may increase the risk of IHD and vascular dysfunction, including processes related to atherosclerosis development, sometimes with dose–response relationships and sex-specific patterns. Mechanistic evidence supports this link, indicating that PFAS exposure induces molecular and cellular alterations relevant to cardiovascular pathophysiology, including increased oxidative stress and vascular inflammation, and disruption of lipid metabolism. In addition, PFAS may affect epigenetic regulation, telomere length, and mitochondrial DNA copy number, which are emerging biomarkers associated with atherosclerosis and IHD and may indicate early cardiovascular vulnerability. Future research integrating innovative approaches and advanced analytical techniques may help address current knowledge gaps and clarify the mechanistic pathways linking PFAS exposure to clinical cardiovascular outcomes.

## 1. Introduction

Cardiovascular disease (CVD) has persisted as a major public health burden over recent decades, consistently ranking among the leading causes of mortality on a global scale [1,2]. In 2022, CVD remained responsible for approximately 32% of all deaths, with the highest impact observed in low- and middle-income countries, as well as among males and older adults [3,4]. Among CVD, ischemic heart disease (IHD) is the leading cause of death, accounting for more than 315 million prevalent cases and approximately 9 million deaths globally in 2022 [5], and its overall disease burden is projected to continue rising through 2050 due to population aging and widening economic disparities [6]. IHD is a chronic, progressive condition characterized by restricted cardiac blood flow. It most often results from coronary heart disease (CHD), which is primarily driven by atherosclerosis, the underlying pathological process that leads to an imbalance between myocardial oxygen supply and demand [2,7]. It may present with a spectrum of manifestations generally referred to as chronic coronary syndromes, or it may evolve into acute clinical entities such as unstable angina and myocardial infarction (MI) [7,8].

Recent analyses conducted across 204 countries and territories from 1990 to 2021 identified metabolic risk factors as accounting for approximately 52% of the IHD burden in high-socioeconomic index (SDI) regions [9,10]. High systolic blood pressure, elevated low-density lipoprotein (LDL) cholesterol, high fasting plasma glucose, and increased body mass index (BMI) together contributed to roughly half of the total disease load [10]. Conversely, in low-SDI regions, behavioral risks (e.g., smoking) and environmental exposures (e.g., air pollution) constitute a larger share of the IHD burden compared with metabolic risks [10].

Atherosclerosis, the primary cause of IHD, progresses from the lipid-streak phase—characterized by lipid accumulation within the intima, foam-cell formation, and macrophage infiltration—to the fibrous plaque phase, marked by the development of a fibrous cap composed of vascular smooth muscle cells [11]. Rupture of this cap can expose the underlying necrotic core of foam cells to the bloodstream, ultimately leading to thrombosis and, consequently, MI and stroke [11,12].

While dyslipidemia has long been recognized as a central driver of atherosclerosis—together with hypertension, obesity, diabetes mellitus, chronic kidney disease, inadequate diet, and sleep deprivation—accumulating evidence suggests that exposure to endocrine-disrupting chemicals (EDCs) may also contribute to disease development [11,12,13,14]. EDCs, which are widely distributed across environmental media, comprise a group of compounds capable of interfering with hormone homeostasis even at low doses, thereby contributing to a range of hormone-mediated biological alterations [15]. Per- and polyfluoroalkyl substances (PFAS), widely used over the past decades in industrial application—including firefighting foams, solvents, personal care products, textile-protective coatings (e.g., Gore-Tex), non-stick cookware (e.g., Teflon), and food-packaging materials—are persistent and bioaccumulative synthetic chemicals, resulting in measurable PFAS concentrations across multiple human biological matrices [15,16,17]. Toxicological and epidemiological studies have linked PFAS exposure to a range of adverse health effects, including reproductive and developmental toxicity, immunosuppression, thyroid dysfunction, liver and kidney disease, and cancer [18]. Furthermore, given the positive associations between serum PFAS levels and hypercholesterolemia, hypertriglyceridemia, and insulin resistance [19,20,21,22], it is reasonable to hypothesize that PFAS may contribute to atherosclerosis, thereby representing a potential novel risk factor for IHD. Conversely, findings from human studies examining the relationship between PFAS exposure and CVD remain somewhat inconsistent [21].

In this narrative critical review, we examine the current evidence on the association between PFAS exposure in both the general population and occupational settings and the risk of IHD, as well as markers of atherosclerosis representing earlier stages of the disease process, discuss the plausible biological mechanisms underlying this relationship, and outline the implications and future strategies for the primary prevention of one of the most impactful diseases worldwide.

## 2. Materials and Methods

This narrative review is based on a comprehensive literature search conducted in PubMed. Relevant studies were identified using combinations of keywords related to PFAS exposure and cardiovascular outcomes, such as “PFAS”, “per- and polyfluoroalkyl substances”, “ischemic heart disease”, “coronary heart disease”, “atherosclerosis”, “cardiovascular disease”, “oxidative stress”, and “epigenetics”.

The search considered studies published up to March 2026, with no strict lower date limit, in order to capture both early and recent evidence. Additional articles were identified through manual screening of reference lists of relevant publications. Studies were selected based on their relevance to the association between PFAS exposure and IHD or related cardiovascular endpoints, including both clinical outcomes and intermediate biomarkers. Priority was given to epidemiological and experimental studies providing original data, as well as to high-quality reviews for contextual interpretation.

Given the narrative nature of this review, no formal systematic inclusion/exclusion criteria or meta-analytic procedures were applied; however, efforts were made to include studies representing diverse populations, exposure settings, and methodological approaches.

## 3. Per- and Polyfluoroalkyl Substances: An Overview

**Figure 1:**
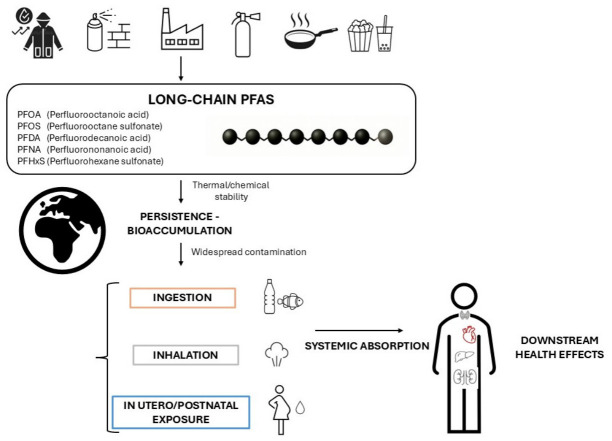
Lifecycle of long-chain per- and polyfluoroalkyl substances (PFAS), illustrating their production, environmental distribution, human exposure pathways (e.g., drinking water, food, air, and dust), bioaccumulation in biological matrices, and potential links to adverse health effects, including cardiovascular outcomes.

PFAS, a diverse group of synthetic chemicals used across multiple industrial sectors (e.g., agriculture, chemical manufacturing, electronics, military applications, packaging, and textiles), are characterized by one of the strongest bonds in chemistry—namely the carbon–fluorine single bond covalently attached to the alkyl chain—and are therefore considered highly persistent environmental contaminants [23,24]. Owing to their extensive use for more than 80 years across over 200 applications, these substances are commonly referred to as “forever chemicals” and are now ubiquitously detected in the environment [23,25]. Their accumulation in environmental media—either directly or via sewage from electroplating plants, laundries, dry cleaners, and household products—is projected to reach 4.4 million tons over the next 30 years unless production is substantially reduced [26,27]. While more than 4000 PFAS have been manufactured and hundreds detected in environmental samples, only a subset—classified as long-chain PFAS (those with more than seven fully fluorinated carbon atoms), such as perfluorooctanoic acid (PFOA), perfluorooctane sulfonate (PFOS), perfluorodecanoic acid (PFDA), perfluorononanoic acid (PFNA), and perfluorohexane sulfonate (PFHxS)—has received the greatest attention due to historically high production volumes and exceptionally long biological half-lives in humans, which result in substantial bioaccumulation even under conditions of chronic low-level exposure [28,29,30]. These compounds can bioconcentrate in aquatic organisms through binding to proteins in the blood, liver, and kidney and consequently biomagnify along associated food webs, posing human health hazards [27,31]. Human exposure occurs primarily through consumption of contaminated drinking water and seafood, particularly in populations residing near polluted sites, followed by inhalation of air and indoor dust and, to a lesser extent, dermal contact [28,32]. Importantly, PFAS can cross the placental barrier and be transferred to infants through breastfeeding, potentially affecting sensitive stages of human development [33,34]. As illustrated in Figure 1, PFAS undergo complex environmental transport and bioaccumulation processes, ultimately leading to multiple human exposure pathways and potential systemic health effects.

Reported maximum PFAS concentrations in drinking water reached 47 ng/L in the Netherlands [35], 60.4 ng/L in China [36], and up to 618 ng/L in the United States [37]. A recent global study of 212 marine fish species sampled between 2010 and 2021 reported a median concentration of C8 PFAS (PFOA + PFOS) of 0.34 ng/g wet weight, with Asia and Oceania displaying the highest levels and cod- and herring-like species contributing most to dietary intake due to their widespread consumption [38]. PFOS exposure exceeded PFOA by approximately one order of magnitude [38].

Such exposure levels have been associated with developmental, hepatic, renal, and hormonal, immunotoxic, carcinogenic and cardiometabolic disorders [18,23,26,39,40]. Human biomonitoring has detected PFOS and PFOA in nearly all individuals assessed, confirming their ubiquitous exposure [28,32,41]. Following ingestion, PFAS are rapidly absorbed in the gastrointestinal tract and preferentially accumulate in high-protein tissues such as the liver and blood. Nonetheless, they can also be detected in several other organs, including the lungs, bone, brain, kidney, and even in breast milk [42]. Most PFAS exhibit slow elimination kinetics in humans, with geometric mean serum half-lives of 3.5 years for PFOA and 4.8 years for PFOS [43], Nonetheless, median serum concentrations vary markedly across geographic regions, reflecting differences in exposure levels, duration, and degrees of industrialization [17]. Besides urinary excretion, the low elimination rate of PFAS is partly attributable to albumin-mediated renal reabsorption [42]. Excretion also occurs via feces and, in women, through parity, lactation, and menstruation, potentially explaining the higher levels of some PFAS in males than in females [42,44]. Therefore, while human biomonitoring of PFAS is typically based on total blood, plasma, or serum, as well as human milk, other non-invasive matrices—such as urine, hair, and nails—can represent valuable tools [45]. These matrices are particularly useful for assessing short-chain PFAS and occupational exposure (urine), PFAS with higher molecular weights (nails), and especially PFOA and PFOS (hair), although data for hair and nails remain limited and highly variable across studies due to differences in population characteristics, exposure sources, and geographic regions [45].

### Legislation of Per- and Polyfluoroalkyl Substances

**Figure 2:**
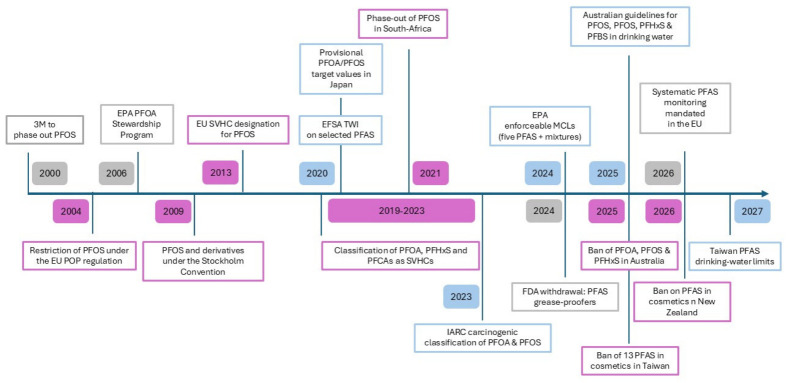
Regulation actions of per- and polyfluoroalkyl substances. Colors indicate the nature of each regulatory event: pink denotes bans and restrictions; light blue represents health-based limits, guidelines, or major toxicological evaluations; gray marks policy, regulatory programs, or industry actions. Abbreviations: EFSA: European Food Security Agency; EPA: Environmental Protection Agency; FDA: Food and Drug Administration; IARC: International Agency for Research on Cancer; MCL: maximum contaminant level; PFBS: perfluorobutane sulfonic acid; PFCA: perfluorocarboxylic acid; PFHxS: perfluorohexane sulfonate; PFOA: perfluorooctanoic acid; PFOS: perfluorooctane sulfonic acid; POP: persistent organic pollutant; SVHC: substance of very high concern; TWI: tolerable weight intake.

While PFAS were considered inert and harmless until around 2000, increasing evidence of their environmental and human health risks led to progressive restrictions on their production (Figure 2) [46]. Early in the current century, the detection of widespread PFOS distribution in wildlife tissues, including albatrosses, bald eagles, polar bears, and seals, raised global concern about its bioaccumulation at higher trophic levels [47]. Consequently, in 2000, 3M—the sole US manufacturer of PFOS—announced the phase-out of PFOS production by the end of 2002 [46,48]. In 2006, the US Environmental Protection Agency (EPA) launched the PFOA Stewardship Program, engaging eight major PFAS-producing companies, including 3M and DuPont, to reduce PFOA emissions and product content by 95% by 2010 and eliminate them entirely by 2015 [49]. Following these measures, blood concentrations of PFOS and PFOA in the US population aged ≥12 years declined by more than 87% and 74%, respectively, between 1999 and 2000 and 2017–2020, although detectable levels persist in most individuals, including those born after the phase-out [50]. Geometric mean concentrations of PFHxS and PFNA also decreased, by 52% and 16%, respectively, albeit more slowly due to their longer half-lives and later regulatory action [51].

In early 2024, the Food and Drug Administration (FDA) announced that PFAS-containing grease-proofing agents were no longer marketed in the United States [49]. In January 2025, the FDA further declared 35 food contact notifications for such PFAS uses in paper and paperboard packaging ineffective, reflecting their complete abandonment [51]. In parallel, the EPA established legally enforceable maximum contaminant levels (MCLs) for PFOA, PFOS, PFHxS, PFNA, and hexafluoropropylene oxide dimer acid (GenX chemicals) in drinking water, with limits ranging from 4 to 10 ng/L, including MCLs for mixtures of at least two of these PFAS [52].

Within the European Union (EU), restrictions on PFOS production and commercialization were first introduced in 2004 under the Persistent Organic Pollutants (POPs) Regulation [53], later replaced by Regulation (EU) 2019/1021, which banned the manufacture and marketing of PFOS and its derivatives [54]. In 2013, the European Chemicals Agency identified PFOA and five other substances as substances of very high concern (SVHCs) due to their persistence, mobility, toxicity, and potential reproductive, mutagenic, and carcinogenic effects [55]. Based on a combined exposure assessment of PFOA, PFNA, PFHxS, and PFOS—accounting for approximately 46% of total PFAS exposure—the European Food Safety Authority (EFSA) reported that toddlers and young children experience mean intake levels nearly twice those of older age groups owing to higher food consumption per kilogram of body weight [56]. Accordingly, EFSA established a tolerable weekly intake (TWI) of 4.4 ng/kg bw (0.63 ng/kg bw per day) for the sum of these four PFAS in 2020 [56]. As estimated mean lower-bound exposures in adolescents and adults range from 3 to 22 ng/kg bw per week, the upper values exceed the TWI by up to a factor of five [56]. In 2023, the Forever Pollution Project identified nearly 23,000 confirmed PFAS-contaminated sites (≥10 ng/L) and over 21,000 presumptive sites across Europe [57]. Since January 2026, EU member states are required to systematically monitor PFAS in drinking water under Directive (EU) 2020/2184, which established limits of 0.5 µg/L for total PFAS and 0.1 µg/L for PFAS of high concern, including mandatory reporting of exceedances and incidents [58,59].

Globally, PFOS and its derivatives have been listed under the Stockholm Convention since 2009, mandating their elimination [60]. This was followed by bans on PFOA in 2019, PFHxS in 2022, and long-chain perfluorocarboxylic acids in 2025, including their salts and derivatives [60]. In November 2023, the International Agency for Research on Cancer classified PFOA as a Group 1 carcinogen and PFOS as Group 2B [61].

As restrictions on long-chain PFAS intensified, industry shifted toward short-chain PFAS (4–7 fully fluorinated carbon atoms), such as PFBS, PFHxA, and related compounds, initially considered safer due to their reduced bioaccumulation potential [31]. However, emerging evidence suggests that their greater water solubility, lower sorption, and higher mobility facilitate widespread environmental dispersion and are associated with cytotoxicity and potential developmental effects [31,62]. Consequently, under the EU REACH framework, PFBS, PFHxA, and 2,3,3,3-tetrafluoro-2-(heptafluoropropoxy)propionic acid were classified as SVHCs between 2019 and 2023 [63,64] (Figure 2).

Referring to other world regions, in June 2025 the Australian National Health and Medical Research Council updated guideline values for PFOA (200 ng/L), PFOS (8 ng/L), PFHxS (30 ng/L), and PFBS (1000 ng/L) to reduce health risks from exposure to these chemicals in drinking water [65]. In the same year, Australia also implemented a nationwide ban on the manufacture, import, export, and use of PFOA, PFOS, and PFHxS [66]. Moreover, the New Zealand Environmental Protection Agency has implemented a ban on PFAS in cosmetics, which entered into force in January 2026 [67].

In Taiwan, regulatory limits will apply from 1 July 2027, requiring that the combined concentration of PFOA and PFOS does not exceed 50 ng/L, and that of PFOS and PFHxS does not exceed 70 ng/L [68]. Additionally, since January 2025, the inclusion of 13 PFAS in the List of Ingredients Prohibited in Cosmetic Products has been in effect [68]. In Japan, from June 2026, PFHxS-related substances will be subject to a ban covering relevant imports and exceptional uses, while the provisional target value of 50 ng/L for total PFOS and PFOA in drinking water, established in 2020, is scheduled to be elevated from April 2026 [69].

In Africa, the regulatory framework on PFAS remains fragmentary with the exception of South Africa, which established the phase-out of PFOS and its salts by the end of 2021 [70].

## 4. The Association Between Exposure to Per- and Polyfluoroalkyl Substances and Risk of Ischemic Heart Disease: The Epidemiological Evidence

To date, only a limited number of epidemiological studies have examined the relationship between PFAS exposure and IHD risk in the general population, mostly within a narrow set of geographic regions. Even fewer investigations have assessed the potential contribution of PFAS to atherosclerosis. Additional studies conducted among PFAS-exposed workers—most employed before progressive regulatory restrictions—have evaluated IHD incidence and mortality, but findings remain inconsistent. Nonetheless, a substantial body of epidemiological and experimental evidence suggests that elevated serum PFAS concentrations may be associated with increased cardiovascular risk [71]. The following sections summarize the main findings from epidemiological studies. Table 1, Table 2 and Table 3 provide a structured overview of individual study findings and limitations; their interpretation should be considered in conjunction with the comparative perspective developed in the text.

### 4.1. Occupational Exposure

The impact of occupational PFAS exposure on IHD was first assessed by Leonard et al. [72] in a cohort of 6027 DuPont Washington Works (WW) employees (1948–2002), (with a median serum PFOA concentration of 494 ng/mL measured among workers in 2004, [73]), where ammonium perfluorooctanoate (APFO) was used as a surfactant. Mortality among WW workers was compared with three reference populations to minimize the healthy worker effect (HWE), a potential bias that systematically leads to an underestimation of occupational health risks when workers are compared with the general population. IHD mortality was significantly lower than expected relative to the US and West Virginia populations (standardized mortality ratio, SMR = 81.2; 95%CI: 71.2–92.1 and SMR = 68.7; 95%CI: 60.2–77.9, respectively), and was not elevated when compared with mortality rates in the DuPont regional worker population (SMR = 109.5; 95%CI: 96.1–124.4). These SMR-based comparisons therefore provide little evidence of an increased IHD mortality risk associated with occupational PFAS exposure [72]. A subsequent retrospective cohort of 4747 WW employees (1947–2002) reported that IHD accounted for 30.9% of all deaths, 63% due to MI [74]. To reduce HWE, the authors used lagged exposure metrics. Across quartiles of cumulative APFO exposure—defined either by IHD cases or by the full cohort—no significant increase in IHD mortality emerged, except for a borderline trend (*p* = 0.06) in the two highest exposure categories with a 10-year lag when quartiles were based on the entire cohort [73,74]. An updated mortality analysis including 5791 DuPont workers with follow-up through 2008 (average annual serum PFOA of 350 ng/mL) again found no significant elevation in overall mortality or positive exposure–response trends for IHD, even with 10- or 20-year lags [75]. In contrast to Sakr et al. [74], SMRs across quartiles showed no increasing trend [75]. A retrospective mortality study of 3993 3M workers exposed to APFO for ≥6 months found no increased IHD mortality (SMR = 0.8; 95%CI: 0.5–1.4) [76]. Cox models showed no association between exposure intensity (median serum PFOA of 2600–5200 ng/mL) or duration and IHD risk after adjustment for confounders. The relatively young cohort and limited deaths were noted as limitations [76].

A longitudinal study of 32,254 subjects—3713 DuPont workers and 28,541 community residents exposed for ≥12 years through contaminated drinking water—evaluated modeled PFOA exposure and incident IHD [77]. The median serum PFOA at baseline was 26.1 ng/mL [77]. Retrospective analyses showed no association between cumulative exposure quintiles and incident IHD in the combined or community cohorts, and patterns of association for MI were comparable [77]. Only males aged 20–39 years showed higher IHD risk in higher exposure quintiles, but without a significant trend [77]. Prospective analyses also showed no exposure-related increase in IHD [77]. The lack of association may reflect treatment for hypercholesterolemia or survivor bias due to the inclusion criteria [77]. A retrospective cohort of 3713 workers, including 1881 with measured serum PFOA in 2005 (median 112.7 ng/mL), evaluated cumulative exposure and incident CHD [78]. Among 399 validated CHD cases (1951–2011), Cox models showed no significant exposure–response trends, with or without a 10-year lag, consistent with the absence of trends for high cholesterol [78]. Because workers had substantially higher exposures, quartile cut-points were correspondingly higher, which may have influenced the observed trends [78].

In sum, published studies do not provide convincing evidence of an association between serum PFOA and increased IHD mortality, aside from a suggestive trend among the most highly exposed workers with a 10-year lag. Similarly, studies of IHD incidence in workers, community residents, and mixed cohorts have not demonstrated a positive association with PFOA exposure, despite consistent links between higher exposure and hypercholesterolemia. These limited and inconsistent findings likely reflect methodological challenges typical of retrospective designs, including imprecise exposure reconstruction—particularly for deceased workers—and highlight the need for future studies with improved exposure assessment and validated cardiovascular endpoints (Table 1).

**Table 1:** Key findings and evidence gaps on exposure to per- and polyfluoroalkyl substances and ischemic heart disease in occupational settings.

References	Pitfalls	References	Clues
[72,74,75,76,78]	Retrospective studies	[72]	Non-significant increase in IHD mortality among WW workers exposed to APFO compared to the regional employee population from the same company
[72]	Significant decrease in SMR mortality for IHD among WW workers in comparison to the US and West Virginia populations	[73]	A significant trend of increasing IHD mortality risk across higher APFO exposure categories observed only at the 10-year lag
[72]	Potential underestimation of exposure for workers who died before 1957		
[74]	No additional significant risk estimates found in any higher exposure category compared with the reference group, nor at any other exposure lags.		
[74]	No information available on individual risk factors for CVD or medication use		
[74,78]	Possibility of selection bias		
[75]	No exposure–response trend observed for IHD mortality in both 10-year- and 20-year-lag analyses		
[76]	No increase in IHD mortality among employees at 3M Company’ Minnesota manufacturing plant exposed to APFO		
[76,77,78]	Potential for misclassification of exposure, disease and covariates		
[76]	Limited smoking data		
[76]	Relatively small number of deaths during the follow-up period		
[77]	No association between PFOA exposure and incident IHD in either the combined community and worker cohorts or the community cohort		
[78]	No association between PFAS exposure (occupational and non-occupational) and incident CHD		
[77,78]	Underestimation of CHD incidence due to exclusion of deceased workers and the requirement to be alive in 2005–2006.		
[77]	Possibility of decreasing susceptibility to PFOA effects with increasing follow-up time		

Abbreviations: APFO: ammonium perfluorooctanoate; CHD: coronary heart disease; CVD: cardiovascular disease; PFAS: per- and polyfluoroalkyl substances; PFOA: perfluorooctanoic acid; SMR: standardized mortality ratio; WW: Washington Works plant facility in Parkersburg.

### 4.2. Exposure in the General Population: Risk of Ischemic Heart Disease

#### 4.2.1. United States

An early investigation assessed the health status of 599 adults recruited through media announcements from a source population of approximately 70,000 residents living near the DuPont plant in West Virginia and exposed to contaminated drinking water [79]. Exposed subjects showed a significantly higher prevalence of angina and MI (Standardized Prevalence Ratio, SPR = 8.07, 95%CI: 6.54–9.95; and SPR = 1.91, 95%CI: 1.40–2.62) compared with NHANES 2001–2002 [79]. However, the absence of an unexposed comparison group, potential self-selection, and the use of contaminated water as a proxy for individual exposure may have introduced bias and exposure misclassification [79]. Using NHANES 1999–2000, 2003–2004 and 2005–2006 data (3974 adults), Melzer et al. [80] examined associations between serum PFOA/PFOS and thyroid disease and other outcomes. Mean serum PFOA and PFOS were higher in men (4.91 vs. 3.77 ng/mL and 25.08 vs. 19.14 ng/mL, respectively) [80]. After adjustment for major confounders, neither compound was significantly associated with CHD, MI, or angina [80]. Despite the long half-life of PFAS, reliance on a single serum measurement may have underestimated exposure [80]. A subsequent study [81] merging NHANES 1999–2000 and 2003–2004 (1327 adults ≥ 40 years) evaluated serum PFOA in relation to CHD, stroke, and peripheral artery disease (PAD), the latter characterized by chronic progressive accumulation of atherosclerotic lesions leading to varying degrees of arterial obstruction and ischemic symptoms in the affected extremities [82]. Increasing PFOA across sex-specific quartiles (<2.9 to >5.6 ng/mL in women; <3.1 to >6.1 ng/mL in men) was significantly associated with combined CHD/PAD prevalence (Odds Ratio, OR = 2.28, 95%CI: 1.40–3.71) [81]. Participants in the highest quartile had higher prevalence of CHD (OR = 2.24, 95%CI: 1.02–4.94) and PAD (OR = 1.78, 95%CI: 1.03–3.08), independent of major confounders [81]. Associations were consistent across sex, BMI categories, and smoking status, except for current smokers, likely due to small numbers; however, the cross-sectional design limits causal inference [81]. A 2012–2013 biomonitoring study of 154 male anglers ≥ 50 years found no significant associations between serum levels of seven PFAS (perfluoroheptane sulfonate, PFDA, PFHxS, PFNA, PFOA, PFOS, and PFuDA) and self-reported CHD or combined CVD, after adjustment for age, BMI, work status, and alcohol consumption, despite modest associations of ΣPFAS and Σsulfonates with high cholesterol [83]. PFAS levels were similar to the US population, except for PFOS, which was nearly twice as high (19 vs. 10 ng/mL) [83]. Consistently, in a cross-sectional study of 5270 adults ≥ 20 years with diabetes from the C8 Health Project, Honda Kohmo et al. [84] reported significant inverse associations between serum PFOA, PFOS, PFHxS (but not PFNA) and CHD prevalence (adjusted ORs 0.72–0.90). A stronger inverse gradient was observed across exposure quintiles [84]. A post hoc analysis in 49,161 nondiabetic individuals confirmed similar inverse associations (ORs 0.92–0.95) [84]. Results were not explained by kidney function because, although 22.2% of participants had chronic kidney disease (CKD), findings were consistent across CKD strata [84]. Using NHANES 1999–2014 (10,859 adults), Huang et al. [85] reported borderline associations between increasing serum PFAS and CHD and MI prevalence (p-trend = 0.0623 and 0.0636). Significant positive associations emerged at higher exposure levels for PFNA, PFDA, and perfluoroundecanoic acid (PFUnDA) with CHD (p-trend = 0.0101, 0.0107, and 0.0008, respectively), for PFNA with MI (p-trend = 0.0240), and for PFUnDA and perfluorododecanoic acid (PFDoA) with angina (p-trend = 0.0408 and 0.0138, respectively) [85]. Only four PFAS (PFOS, PFOA, PFNA, and PFHxS) were detected in >98% of participants, while others had detection frequencies of 30–65% [85]. The lack of associations for PFOS and PFOA likely reflects declining exposure due to phase-out, although participants with CVD had higher total PFAS and PFOS levels [85]. In NHANES 2003–2012 (7904 adults), Feng et al. [86] found that a log-unit increase in PFOS was weakly associated with MI among males (OR = 1.01, 95%CI: 1.00–1.01), and a log-unit increase in PFNA was associated with a 10% higher risk of CHD and MI (p = 0.022 and 0.028). These sex-specific findings may reflect faster PFAS elimination in women through menstruation, pregnancy, and lactation [86]. Finally, in two nested case–control studies within the Health Professionals Follow-up Study and Nurses’ Health Study cohorts, Zhu et al. [87] evaluated participants initially free of CVD who later developed CHD (n = 101). After adjustment for major confounders, higher plasma total PFOS, branched PFOS, and linear PFOS were significantly associated with increased CHD risk (OR = 3.66, 3.68, and 3.01, respectively), with clear dose–response relationships [87]. No significant associations were observed for PFOA, PFDA, PFNA, or PFHxS [87]. These PFOS–CHD associations do not appear mediated by lipid pathways, given the absence of associations with apolipoprotein (apo)C-III levels, a key predictor of cardiovascular outcomes [88]. Conversely, positive associations of PFDA, PFNA, and PFHxS with apoE-containing HDL particles may help explain their non-significant inverse associations with CHD [87].

#### 4.2.2. Europe

Within a longitudinal setting, Mattsson et al. [89] recruited 253 men with a CHD diagnosis between 1992 and 2009, identified through national registers, along with age-matched controls. All participants provided a baseline blood sample in 1990–1991, and 104 case–control pairs contributed a second sample in 2002–2003 for measurement of eight PFAS. Serum PFOS and PFOA significantly decreased over time, whereas PFDA, PFNA, and PFHxS increased; perfluoroheptanoic acid (PFHpA), PFUnDA and PFDoA showed no significant temporal changes. Unlike [85], no differences in PFAS were observed between cases and controls, suggesting that CHD does not substantially influence PFAS levels [89]. Considering exposure at baseline, no PFAS was significantly associated with CHD risk except PFHpA, whose third quartile showed a higher risk (OR = 2.58, 95%CI: 1.39–4.78) than the first quartile [89]. The authors considered this finding likely due to chance, given the much lower PFHpA concentrations relative to those of PFOA [89]. On the other hand, the exclusive inclusion of men may also have introduced sex-related bias due to sex-specific PFAS toxicokinetics [89]. More recently, Schillemans et al. [90] conducted a nested case–control study using two Swedish cohorts: the SMC C (women in Uppsala) and the 60YO cohort (men and women in Stockholm), with baseline samples collected between 2003 and 2009 and 1997–1998, respectively. During follow-up (to 2017 for SMC C and 2014 for 60YO), 345 incident MI cases were identified and matched to controls (2:1 in SMC C; 1:1 in 60YO; total 475 controls) [90]. The highest tertile of the PFAS sum (eight compounds) and most individual PFAS (PFOS, PFNA, PFDA, and PFUnDA) was significantly associated with higher total and LDL cholesterol [90]. At the same time, most PFAS showed favorable metabolic associations, including higher HDL cholesterol and apoA1, the major protein component of HDL and a marker linked to reduced atherosclerotic risk [91], as well as lower triglycerides [90]. After full adjustment, ∑PFAS was inversely associated with CVD risk in the pooled cohorts (OR = 0.73; 95%CI: 0.55–0.97) [90]. A similar inverse pattern emerged for MI (pooled OR = 0.60; 95%CI: 0.39–0.92) [90]. Thus, despite the established relationship between LDL cholesterol and MI [92,93], and despite PFAS-related increases in LDL, this did not translate into higher MI risk, possibly due to the counterbalancing favorable effects of PFAS on HDL cholesterol, apoA1, and triglycerides, which may attenuate or outweigh LDL-related effects [90]. Alternatively, PFAS-induced LDL increases may be insufficient to raise CVD risk, consistent with the lack of association between PFAS (combined or individual) and apoB, a stronger atherogenic marker than LDL cholesterol [90,94]. Finally, the inability to include PFOA and PFHpA in SMC C analyses due to sample contamination may have led to an underestimation of PFAS effects [90].

#### 4.2.3. China

A hospital-based case–control study including 355 newly diagnosed ACS patients (MI or unstable angina) aged 18–75 years and 466 age- and sex-matched controls evaluated associations between six plasma PFAS and the risk of acute coronary syndrome (ACS, including MI and instable angina) [95]. In fully adjusted models, only PFOA (OR = 2.43, 95%CI: 1.34–4.39, p = 0.006), PFOS (OR = 1.65, 95%CI: 1.14–2.38, p = 0.013), and PFUnDA (OR = 1.50, 95%CI: 1.07–2.09, p = 0.024) were significantly associated with ACS [95]. In multiple-PFAS models, significant associations persisted only for PFOA and PFOS (OR = 1.51, 95%CI: 1.07–2.15, p = 0.049; OR = 1.77, 95%CI: 1.15–2.72, p = 0.034), likely reflecting their much higher plasma concentrations—up to sevenfold higher than those of other PFAS [95]. The overall mixture effect was null, as only PFOA, PFOS, and PFUnDA contributed positively [95]. In sex-stratified analyses, no associations were detected in females, whereas PFOA, PFOS, PFHxS, and PFDA were significantly associated with ACS in males, likely due to lower PFAS levels and a lower prevalence of ACS in women [95]. Despite inverse associations of platelet count, mean platelet volume, and plateletcrit (the product of mean platelet volume and platelet count)—and a positive association of platelet distribution width—with ACS risk, PFOS was inversely associated only with platelet count, which attenuated its ACS effect by >15%, suggesting a potential mechanistic pathway [95].

A second study of 571 consecutive ACS patients aged 18–80 years with no occupational PFAS exposure assessed whether PFAS influence baseline coronary stenosis and ACS prognosis [96]. Coronary stenosis was quantified using the Gensini score (GS) and the number of lesioned vessels (LVN), and prognosis evaluated through MACE (cardiovascular death, nonfatal MI, stroke, and revascularization) over a median 14.3-month follow-up [96]. Plasma PFOS was significantly associated with GS (Hazard Ratio, HR = 1.33, 95%CI: 1.06–1.67, p = 0.034) and LVN (HR = 1.36, 95%CI: 1.08–1.71, p = 0.023) after full adjustment [96]. PFOS and total PFAS were also positively associated with MACE (HR = 1.96, 95%CI: 1.34–2.89, p = 0.002; HR = 2.46, 95%CI: 1.51–4.01, p = 0.002) and nonfatal MI (HR = 3.86, 95%CI: 2.00–7.46, p = 0.000; HR = 4.56, 95%CI: 1.99–2.45, p = 0.001), with similar results for highest tertile comparisons [96]. Threshold PFOS concentrations were identified for each endpoint (4.65 ng/mL for GS; 4.54 ng/mL for LVN; 5.14 ng/mL for MACE; and 5.03 ng/mL for nonfatal MI), with variability across compounds reflecting structural and toxicokinetic differences [96]. Inconsistent PFOA effects relative to [95] may reflect lower PFOA levels in this cohort (4.39 vs. 4.99 ng/mL) [96]. Furthermore, the absence of associations between PFAS mixtures and stenosis severity or revascularization may relate to differential susceptibility of these indicators and heterogeneous mixture effects [96].

Overall, current evidence regarding the contribution of PFAS exposure to the occurrence of IHD in the general population remains inconsistent. However, suggestive findings indicate associations with certain PFAS compounds, particularly PFOS and PFOA, which, despite regulatory restrictions, have historically reached the highest environmental levels and contributed most to human exposure. The predominantly cross-sectional design of most published studies, together with the lack of repeated PFAS measurements, prevents firm conclusions about causality, even when significant positive associations are observed. Importantly, because most available studies are cross-sectional and rely on single PFAS measurements, they cannot establish whether PFAS exposure preceded the development of atherosclerosis or whether lifestyle or physiological changes associated with existing CVD may have influenced PFAS distribution and excretion. Moreover, differences in study populations (often male-only and largely US-based), limited sample sizes, heterogeneous confounder adjustment, and the variety of congeners examined further complicate comparisons. Future longitudinal investigations across diverse geographic areas, including populations exposed to levels exceeding background concentrations and incorporating comprehensive adjustment for established cardiovascular risk factors, are needed to strengthen the current evidence base (Table 2).

**Table 2:** Key findings and evidence gaps on exposure to per- and polyfluoroalkyl substances and ischemic heart disease in the general population.

References	Pitfalls	References	Clues
[79,81,83,84,85,86]	Self-reported diseases/symptoms	[79]	PFOA exposure via drinking water significantly associated with increased self-reported prevalence of angina and MI
[79]	Potential selection bias	[81]	Increasing PFOA concentrations significantly associated with a higher prevalence of CHD and PAD, independent of traditional cardiovascular risk factors
[79]	No true unexposed control group for comparison	[85]	Signals of significant positive association between the highest exposure to the PFAS sum and the prevalence of CHD and MI
[79]	Potential exposure misclassification	[85]	Increasing quartiles of serum PFNA, PFDA, and PFUnDA significantly and positively associated with the prevalence of CHD
[79]	No adjustment for confounders	[85]	Increasing quartiles of serum PFNA significantly and positively associated with the prevalence of MI
[80]	No significant association between serum PFOA and PFOS concentrations and the prevalence of IHD	[85]	Increasing quartiles of serum PFUnDA and PFDoA significantly and positively associated with the prevalence of angina pectoris
[80,81,83,84,85,86,87,90,95,96]	Single PFAS measurement	[86]	Log-unit change in PFOA levels modestly but significantly associated with increased MI risk among males
[13,80,81,83,84,85,86,87,95,96]	Cross-sectional design	[86]	Log-unit change in PFNA levels significantly associated with increased MI and CHD risk among males
[83,89]	No significant association between serum levels of any of PFAS examined and the risk of CHD	[87]	Higher plasma levels of total PFOS, branched PFOS, and linear PFOS significantly associated with an increased risk of CHD
[83,89]	Cohort composed solely of men	[89]	Significant association only between serum levels of PFHpA and a higher risk of CHD for the third quartile compared to the lowest quartile
[84]	Serum PFAS levels significantly inversely associated with CHD in subjects with diabetes independently of the presence of CKD	[90]	Plasma levels of the PFAS sum, PFOS, PFDA, PFNA, and PFUnDA significantly and positively associated with higher concentrations of total and LDL cholesterol
[87]	No significant positive associations between PFAS with lipoprotein subspecies relevant to CHD risk	[95]	Plasma PFOA and PFOS significantly and positively associated with ACS risk
[87]	Serum levels of PFNA, PFDA and PFHxS significantly and positively associated with total apoE among HDL particles with or without apoC-III	[95]	Dose–response relationship with an increasing trend between PFOA and PFOS and ACS risk
[90]	Plasma levels of the PFAS sum, PFOS, PFOA, PFDA, PFNA, and PFUnDA significantly and positively associated with higher concentrations of HDL cholesterol and apoA1	[96]	Significant positive association between plasma PFOS concentration and coronary stenosis severity in patients with ACS
[90]	Plasma levels of the PFAS sum, PFOS, PFOA, PFDA, PFNA, and PFUnDA significantly and positively associated with higher concentrations of HDL cholesterol and apoA1	[96]	Significant positive association between plasma levels of total PFAS and PFOS and the occurrence of MACE in patients with ACS
[90]	Plasma levels of the PFAS sum, PFHxS, PFOS, PFDA, PFNA, and PFUnDA significantly and positively associated with lower concentrations of triglycerides		
[90]	No significant association between plasma levels of PFAS and apoB		
[90]	Plasma PFAS levels significantly and inversely associated with incident MI		
[87,90]	Cross-sectional design of lipid analyses		
[84,86,87,90,95]	Potential residual or unmeasured confounding		
[90]	Possibility of underestimation of MI risk due to the use of lipid-lowering medications during follow-up		
[95]	Plasma levels of PFNA, PFDA, PFHxS and PFUnDA not significantly associated with or inversely correlated with ACS risk		
[95]	No significant association between plasma PFAS mixture levels and ACS risk		
[79,83,87,95]	Limited sample size		
[87]	Homogeneous socioeconomic status among participants		

Abbreviations: ACS: acute coronary syndrome; Apo: apolipoprotein; CHD: coronary heart disease; CKD: chronic kidney disease; HDL: high-density lipoprotein; MI: myocardial infarction; MACE: major adverse cardiovascular effects; PAD: peripheral arterial disease; PFDA: perfluorodecanoic acid; PFDoA: perfluorododecanoic acid; PFHpA: perfluoroheptanoic acid; PFNA: perfluorononanoic acid; PFOA: perfluorooctanoic acid; PFOS: perfluorooctane sulfonic acid; PFUnDA: perfluoroundecanoic acid; PFHxS: perfluorohexane sulfonate.

### 4.3. Exposure in the General Population: Association with Atherosclerosis Development

A limited number of studies have examined the link between PFAS exposure and atherosclerosis, the main contributor to IHD. In a cross-sectional study of 664 Taiwanese individuals aged 12–30 years, Lin et al. [13] assessed PFAS exposure in relation to carotid intima–media thickness (IMT), a validated surrogate of atherosclerosis and a predictor of CHD and MI [97]. Among PFOA, PFOS, PFNA, and PFUnDA, only PFOS showed a significant positive association with IMT (p-trend < 0.001), whereas PFNA was inversely associated [13]. Subgroup analyses confirmed negative associations for PFNA in females, younger participants, those with BMI < 24, non-smokers, and *APOE ε2/ε2* or *ε2/ε3* carriers [13]. *APOE* variants influence CVD risk, with *ε2* generally protective and *ε4* associated with higher IMT and LDL cholesterol [98,99]. The authors suggested that age, sex, obesity, smoking, and *APOE* genotype may exert stronger effects on IMT than PFOS itself [13]. Similar to other EDCs, PFOS also showed a non-monotonic dose–response pattern, with maximal effects at the 50th–75th percentiles [13,100]. Although PFNA was inversely associated with IMT, combined high PFOS (>50th percentile) and low PFNA (≤60th percentile) tripled the risk of thicker IMT (OR = 3.01; 95%CI: 1.68–5.39) [13]. In a subsequent cohort of 848 subjects aged 12–30 years, the same group evaluated PFAS exposure, oxidative stress, and endothelial and platelet microparticles [101]. Endothelial microparticles (CD31+/CD42a−), markers of endothelial injury [101,102], increased across PFOS quartiles (p-trend < 0.001), as did CD31+/CD42a+ (a marker of platelet apoptosis; p = 0.010) and IMT (p < 0.001) [101]. In contrast, CD31+/CD42a− levels were inversely associated with PFOA, PFNA, and PFUnDA [101]. PFUnDA was also inversely associated with CD62E, a marker of endothelial activation, and with IMT [101]. No PFAS was associated with urinary 8-hydroxydeoxyguanosine, a marker of oxidative DNA damage [101,103]. The highest IMT risk occurred when both CD31+/CD42a− and CD31+/CD42a+ exceeded the 50th percentile (OR = 2.86; 95%CI: 1.69–4.84) [101]. As in previous studies, the cross-sectional design limits causal inference. Lind et al. [104] assessed carotid IMT, intima–media echogenicity, and plaque presence in 1016 seventy-year-old Swedish adults. Eight PFAS (PFOA, PFOS, PFDA, PFNA, PFHxS, PFUnDA, PFHpA, and perfluorooctane sulfonamide—PFOSA) were detected in >80% of participants [104]. Unlike Lin et al. [13], no PFAS was associated with IMT in sex-adjusted or sex-stratified models, except PFOSA, which was positively associated with IMT in the fully adjusted model and among females (p = 0.01 and p = 0.004) [104]. Several PFAS showed sex interactions for intima–media echogenicity: PFNA was positively associated among females, whereas PFUnDA was inversely associated among males, with minimal mediation by cholesterol [104]. No PFAS was associated with carotid plaques overall, except PFUnDA, which increased plaque risk among females (OR = 1.59; 95%CI: 1.03–2.43) [104]. Using data from 666 prediabetic adults in the US Diabetes Prevention Program, Osorio-Yáñez et al. [105] evaluated PFAS exposure (six compounds) in relation to coronary artery calcium (CAC) and thoracic aorta calcification (TAC). CAC, quantified by the Agatston score, is a gold-standard marker of subclinical atherosclerosis and a predictor of CHD [106,107,108]. Each doubling of total PFOS and linear PFOS was associated with higher odds of severe CAC (OR = 1.49, 95%CI: 1.01–2.21; OR = 1.54, 95%CI: 1.05–2.50) [105]. N-ethyl-perfluorooctane sulfonamido acetic acid (EtFOSAA) showed a clear dose–response relationship, with each doubling associated with moderate CAC (OR = 1.26, 95%CI: 1.08–1.47) and severe CAC (OR = 1.37, 95%CI: 1.07–1.74) [105]. Total PFOS was also associated with ascending aorta calcification (OR = 1.67, 95%CI: 1.10–2.54), driven by linear PFOS (OR = 1.70, 95%CI: 1.13–2.56) [105]. In contrast, no significant associations were observed between EtFOSAA and TAC [105]. Although PFAS mixtures were associated with moderate–high CAC in crude models, associations were not significant after full adjustment [105].

**Table 3:** Key findings and evidence gaps on exposure to per- and polyfluoroalkyl substances and atherosclerosis in the general population.

References	Pitfalls	References	Clues
[13,101,104]	Single PFAS measurement	[13]	Increasing quartiles of serum PFOS and PFNA and significantly and positively associated with carotid IMT
[13,92,104]	Cross-sectional design	[101]	Significant positive associations between increasing serum PFOS concentrations, CD31+/CD42a− and CD31+/CD42a+ levels, and carotid IMT
[13]	Increasing levels of serum PFNA significantly and inversely associated with carotid IMT	[101]	The most relevant association between PFOS and carotid IMT observed when both levels of CD31+/CD42a− and CD31+/ CD42a+ are elevated
[13]	Most relevant associations between PFOS exposure and carotid IMT observed in younger, non-obese individuals and in those carrying *APOE* ε2 or ε2/ε3 genotype	[104]	Serum PFOSA levels significantly associated with thicker IMT in the whole cohort and among women
[13,101,104,105]	No adjustment for other environmental contaminants and medications	[104]	PFNA exposure significantly and positively associated with IM-GSM among women
[13,101]	Population study consisting of exclusively adolescents and young adults	[104]	PFUnDA significantly associated with an increased number of carotid arteries plaques among females
[13]	Other unknown processes capable of increasing both serum PFOS levels and carotid IMT	[105]	Each doubling of the mean sum of linear and branched isomers of PFOS significantly associated with severe CAC
[101]	Significant inverse associations between increasing serum PFNA levels and CD31+/CD42a− and CD62E levels	[105]	Each doubling of the mean plasma concentration of linear PFOS significantly associated with severe CAC
[101]	Significant inverse relationship between increasing categories of PFOA and PFNA and CD31+/CD42a− levels	[105]	Each doubling of the mean plasma concentration of EtFOSAA significantly associated with CAC in a dose-dependent manner
[101]	No significant association between serum PFAS and urinary 8-OHdG levels	[105]	Each doubling of the mean plasma concentration of PFOS and linear PFOS significantly associated with AsAC
[104]	No significant association between serum PFAS levels and IMT (except for PFOSA) and IM-GSM in the sex-combined analyses		
[104]	Serum PFUnDA concentration inversely associated with IM-GSM among men		
[104]	Sample limited to only Caucasian aged 70 years		
[105]	No significant association between the mean plasma PFAS mixture concentration and CAC		
[105]	No significant association between any PFAS compound and DAC		
[105]	Lack of repeated CAC measurements		

Abbreviations: *APOE*: apolipoprotein E; AsAC: ascendent aortic calcification; CAC: coronary artery calcium; DAC: descendent aortic calcification; EtFOSAA: N-ethyl-perfluorooctane sulfonamido acetic acid; IM-GSM: intima–media gray scale median; IMT: intima–media thickness; PFAS: per- and polyfluoroalkyl substances; PFNA: perfluorononanoic acid; PFOS: perfluorooctane sulfonic acid; PFOSA: perfluorooctane sulfonamide; PFUnDA: perfluoroundecanoic acid.

Collectively, and when considered alongside findings from studies on IHD occurrence, exposure to certain PFAS congeners may contribute to atherosclerosis, as suggested by associations with increased IMT, endothelial and platelet dysfunction, CAC, and thoracic aortic calcification, sometimes with sex-specific patterns. In contrast, findings for other vascular markers, such as intima–media echogenicity and carotid plaques, remain inconsistent.

In this context, some patterns begin to emerge across studies. Positive associations appear to be more consistently reported for certain congeners, particularly PFOS, whereas findings for other PFAS remain heterogeneous. Moreover, associations tend to be more robust in studies using objective or subclinical endpoints, such as IMT and CAC, compared with those relying on self-reported cardiovascular outcomes. Differences in population characteristics, including age, exposure levels, and geographic context, may further contribute to variability in the results.

Evidence remains limited, and most studies rely on cross-sectional designs with single PFAS measurements. Future multicenter prospective studies incorporating repeated exposure measurements and comprehensive vascular biomarkers in larger, multiethnic cohorts are required to clarify these associations. Together, these findings highlight a heterogeneous but suggestive pattern in which associations appear more consistent for intermediate vascular markers than for clinical IHD outcomes.

## 5. Oxidative Stress, Epigenetic and Genetic Modifications Underlying PFAS-Induced Ischemic Heart Disease

Although epidemiological evidence increasingly links PFAS exposure to cardiovascular outcomes, including IHD, the molecular dynamics underlying these associations remain to be fully characterized. Described mechanisms include disturbances in lipid metabolism (e.g., increased total and LDL cholesterol [109]), platelet activation [110], inflammation and endothelial dysfunction [86], cardiomyocyte remodeling [111], elevated blood pressure and arterial stiffness [112], together with oxidative stress, a key driver of PFAS-related cardiovascular toxicity [113].

**Figure 3:**
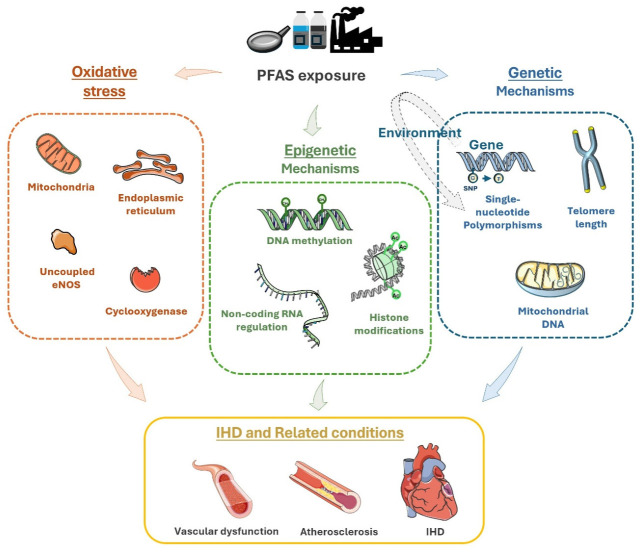
Overview of PFAS-induced molecular mechanisms potentially contributing to ischemic heart disease, including oxidative stress, epigenetic alterations (DNA methylation, histone modifications, and non-coding RNA regulation), and genetic changes such as telomere shortening and altered mitochondrial DNA copy number. Image adapted from Servier Medical Art (https://smart.servier.com, accessed on 20 April 2026), licensed under CC BY 4.0 (https://creativecommons.org/licenses/by/4.0/). Abbreviations: DNA: deoxyribonucleic acid; eNOS: endothelial nitric oxide synthase; IHD: ischemic heart disease; PFAS: per- and polyfluoroalkyl substances; RNA: ribonucleic acid; SNP: single-nucleotide polymorphisms.

Beyond these alterations, more subtle cellular modifications may occur and contribute to IHD risk. Figure 3 summarizes the main molecular mechanisms proposed to link PFAS exposure to IHD risk, integrating oxidative stress, epigenetic regulation, and genetic susceptibility factors. Growing attention has been directed toward epigenetic regulation as a potential mediator of PFAS-induced cardiovascular effects [114]. In parallel, genetic alterations in terms of telomere shortening and variations in mitochondrial DNA copy number (mtDNAcn) have been identified as markers of PFAS effects [115] and have also independently associated with IHD risk [116,117,118]. Emerging evidence further indicates that genetic background may modulate individual susceptibility to PFAS-related effects within a gene–environment interaction framework, thereby contributing to inter-individual variability in disease risk [119].

However, direct and robust evidence linking PFAS-induced epigenetic and genetic alterations to IHD remains limited. Therefore, this review not only summarizes the available studies on oxidative stress in IHD-related conditions and models, but also highlights emerging molecular mechanisms that may underlie the relationship between PFAS exposure and cardiovascular risk. These mechanisms are considered in light of current evidence, which remains incomplete and requires further investigation.

### 5.1. Oxidative Stress: A Key Mechanism in PFAS-Induced Cardiovascular Toxicity

Oxidative stress is a condition characterized by an imbalance between the production of reactive oxygen species (ROS) and the capacity of endogenous antioxidant defense systems. Major sources of ROS in vascular and cardiac cells include mitochondria, uncoupled endothelial nitric oxide synthase (eNOS), cyclooxygenases, and the endoplasmic reticulum. Oxidative stress plays a central role in the pathophysiology of CVD. It promotes lipid peroxidation, protein modification, and DNA damage, ultimately contributing to endothelial dysfunction, atherosclerosis progression, cardiomyocyte death, and adverse cardiac remodeling, which collectively underlie the pathophysiology of IHD [120,121].

Notably, growing evidence indicates that PFAS promotes oxidative stress in cardiovascular tissues, thereby triggering a cascade of events relevant to atherosclerosis and the subsequent development of IHD [113].

PFOS has been shown to induce ROS generation in human microvascular endothelial cells in a concentration-dependent manner. This response is associated with cytoskeletal reorganization and increased endothelial permeability, processes that may compromise vascular integrity and favor early pro-atherogenic changes [122]. ROS production can also be accompanied by hallmarks of endothelial dysfunction, ultimately contributing to endothelial activation, eNOS uncoupling, and enhanced monocyte recruitment, which are key steps in early atherogenesis [123].

More recent mechanistic evidence further supports the link between PFOS exposure and oxidative stress-driven endothelial dysfunction. Using human microvascular endothelial cells, Vajeethaveesin et al. showed that PFOS elicits a broad cellular stress response involving activation of the ER stress-related HRI/eIF2α/ATF4 pathway, a key upstream amplifier of oxidative stress signaling, along with inhibition of SIRT1, a critical regulator of redox homeostasis. This response is accompanied by activation of nuclear factor kappa-light-chain-enhancer of activated B cells and JAK2/STAT3 signaling pathways and by increased expression of key pro-inflammatory and prooxidant mediators, including interleukin 6, intercellular adhesion molecule 1, and COX-2, which play a central role in endothelial activation and atherogenesis. Several of these molecular alterations, such as ATF4, C/EBPβ, and XBP1, have also been identified as upregulated in transcriptomic datasets from patients with atherosclerosis, supporting the translational relevance of PFOS-induced oxidative stress pathways in human cardiovascular disease [124].

Beyond endothelial cells, PFAS exposure also affects other cell types involved in IHD-related processes. Yue et al. reported that PFOS promotes intimal hyperplasia and atherosclerosis by inducing a proliferative and migratory phenotype in human aortic smooth muscle cells. Transcriptomic analyses revealed coordinated changes in pathways related to cell cycle regulation, inflammation, extracellular matrix remodeling, and oxidative stress, consistent with a multifaceted pro-atherogenic response [125].

PFAS exposure typically occurs as complex mixtures rather than individual compounds, which may enhance biological effects. In this context, Zhang et al. showed that combined PFAS exposure induces synergistic cytotoxicity in human-induced pluripotent stem cell-derived cardiomyocytes, significantly reducing cell viability. These effects are associated with mitochondrial dysfunction and altered cellular metabolism, accompanied by increased ROS production and disruption of redox homeostasis [126].

Despite increasing in vitro evidence supporting oxidative stress as a central mechanism of PFAS-induced cardiovascular toxicity, animal studies explicitly addressing this pathway remain limited. In a zebrafish model, PFOSA exposure induced cardiotoxicity through activation of the aryl hydrocarbon receptor, leading to increased oxidative stress, impaired antioxidant defenses, and enhanced lipid peroxidation [127].

Similarly, in a murine model of diet-induced obesity, exposure to PFOS or PFHxS resulted in upregulation of oxidative stress-related targets and enrichment of the circulating lipidome with oxidized lipid species, a recognized hallmark of pro-atherogenic vascular injury [128]. These alterations, together with broader changes in serum phospholipids, sphingomyelins, and triglycerides, support the hypothesis that PFAS-induced oxidative stress contributes to systemic lipid dysregulation, thereby promoting atherosclerosis and increasing IHD risk, particularly under conditions of metabolic stress.

In line with in vitro and animal evidence, emerging human studies further support the association between PFAS exposure and cardiovascular risk through mechanisms involving oxidative stress. Wang et al. provided initial evidence that low-level environmental PFAS exposure is associated with alterations in the human serum metabolome indicative of oxidative and nitrosative stress. Specifically, disruptions in glutathione metabolism, nitric oxide signaling, and mitochondrial pathways were observed, pointing to endothelial dysfunction and leading the authors to suggest a potential increase in cardiovascular risk, including IHD [129].

A cross-sectional study by Lin et al., involving approximately 600 participants, further reported a positive association between serum PFOS levels and increased concentrations of 8-hydroxy-2′-deoxyguanosine, a marker of oxidative DNA damage, along with elevated LDL cholesterol levels [130]. These observations support a potential link between PFAS exposure, oxidative stress, and atherosclerosis-related processes.

### 5.2. Epigenetic Alterations: DNA Methylation, Histone Modifications, and Non-Coding RNAs Regulation

Epigenetic alterations include mechanisms such as DNA methylation, histone modifications, and non-coding RNA regulation, which modulate gene expression without altering the DNA sequence. These processes can affect cellular function and contribute to disease development. Epigenetic changes can be triggered by several endogenous and exogenous factors, including exposure to environmental pollutants such as PFAS [114].

DNA methylation is one of the main epigenetic mechanisms and involves the covalent addition of a methyl group at the 5′ position of cytosine residues within CpG regions by DNA methyltransferases (DNMTs). This modification plays a key role in gene regulation and is generally associated with transcriptional repression. PFAS have been shown to alter both global and gene-specific DNA methylation patterns, either directly or indirectly through mechanisms involving oxidative stress and hormonal imbalance [131].

A large cross-sectional study including 1425 young and middle-aged individuals reported a positive association between serum PFOS levels, increased global DNA methylation, and greater carotid IMT [132].

DNA methylation patterns are also incorporated into mathematical models known as epigenetic clocks, which estimate biological aging and age-related disease risk. Higher serum concentrations of PFNA and PFSA have been associated with accelerated epigenetic aging, as assessed by DNA methylation-based clocks, with stronger effects observed in older individuals and males [133].

Evidence from pregnancy studies further supports the potential long-term impact of PFAS exposure. Maternal exposure to PFAS, particularly PFHxS, has been associated with widespread alterations in placental DNA methylation affecting genes involved in fetal development and cardiometabolic pathways, including lipid metabolism, inflammation, and vascular function [134]. These processes are mechanistically linked to atherosclerosis and, consequently, to IHD risk. Consistently, a longitudinal epigenome-wide association study by Liu et al. reported that gestational exposure to PFOA, PFOS, PFHxS, and especially PFNA was associated with differential DNA methylation at 435 CpG sites in children, many of which map to genes implicated in cardiovascular disease and other major health outcomes [135].

Histone modifications encompass a range of chemical changes—such as acetylation, methylation, phosphorylation, and ubiquitination—that occur mainly on histone tails and regulate gene expression by altering chromatin structure and histone–DNA interactions. Emerging evidence suggests that PFAS may disrupt these regulatory processes either by directly inhibiting enzymes involved in histone modification or by indirectly modulating their activity through endocrine- and oxidative stress-related mechanisms [131]. PFAS exposure has also been shown to affect histone modification dynamics during development. In an in vivo model, maternal exposure to PFOA-induced liver toxicity in offspring, accompanied by reduced histone acetylation and decreased expression of peroxisome proliferator-activated receptor alpha (PPAR-α) [136]. These changes are mechanistically linked to pathways relevant to atherosclerosis and IHD, including impaired lipid metabolism, increased oxidative stress, vascular inflammation, and maladaptive cardiac remodeling [137,138].

Non-coding RNAs represent another important class of epigenetic regulators. Their functions vary depending on the RNA type: microRNAs (miRNAs) bind to target messenger RNAs to inhibit translation or promote degradation; long non-coding RNAs act as scaffolds for chromatin-remodeling complexes or as molecular “sponges” for miRNAs; circular RNAs regulate the intracellular availability of miRNAs. Together, these regulatory networks play a central role in cellular responses to environmental stressors, including PFAS exposure, and may influence disease susceptibility and progression [139].

PFAS exposure has been associated with alterations in specific circulating miRNAs. In particular, PFOS and PFHxS have been linked to the downregulation of miR-101-3p, miR-144-3p, and miR-19a-3p, whose target genes are involved in DNA methylation, lipid metabolism, and inflammatory pathways [140]. Through these mechanisms, miRNA dysregulation may contribute to PFAS-related cardiovascular risk.

Exposure to PFOS and PFHxS has been associated with the downregulation of three serum microRNAs—miR-101-3p, miR-144-3p, and miR-19a-3p—which regulate genes involved in DNA methylation, lipid metabolism, and inflammatory pathways, including DNMT3a, PPAR-α, EGFR (epidermal growth factor receptor), HMGCR (3-hydroxy-3-methylglutaryl-CoA reductase), NR1H3 (nuclear receptor subfamily 1 group H member 3), PTGS2 (prostaglandin–endoperoxide synthase 2), and TGF-α (transforming growth factor alpha) [140]. Through their effects on lipid metabolism, vascular inflammation, and endothelial function, these alterations may contribute to PFAS-induced cardiovascular risk [140]. In addition, studies in two independent pediatric cohorts have shown that PFAS exposure is associated with reduced levels of miR-148b-3p and miR-29a-3p, both implicated in cardiovascular disease pathways and related chronic conditions [141].

### 5.3. Genetic Alterations and Susceptibility: Focus on Telomere Length, Mitochondrial DNA Copy Number, and Single-Nucleotide Polymorphisms

Telomere length and mitochondrial DNA copy number (mtDNAcn) have emerged as informative biomarkers of genomic stability and cellular aging, largely due to their accessibility in peripheral blood. Accumulating evidence indicates that these markers are sensitive to environmental exposures and may reflect the biological impact of external stressors, thereby providing insight into individual susceptibility to disease [142]. Telomeres are composed of repetitive TTAGGG nucleotide sequences located at the ends of linear chromosomes and play a key role in preserving genomic integrity. In eukaryotic cells, telomeres progressively shorten with each cycle of DNA replication due to the “end-replication problem,” ultimately limiting cellular replicative capacity. Telomere attrition is widely regarded as a hallmark of biological aging and cumulative cellular stress and has been associated with several conditions, including IHD [143].

A study including 175 adults aged 50–65 years reported an inverse association between leukocyte telomere length (LTL) and PFOS exposure [144]. In a larger population of 453 individuals, telomere shortening was associated with serum concentrations of PFOA, perfluoro-2,5-dimethyl-3,6-dioxanonanoic acid, and perfluoro-2-methoxyacetic acid [144].

A study recruiting 175 adults aged 50–65 years reported an inverse association between leukocyte telomere length (LTL) and PFOS exposure [144]. In a larger population including 453 individuals, telomere shortening was linked to PFOA, perfluoro-2,5-dimethyl-3,6-dioxanonanoic acid, and perfluoro-2-methoxyacetic acid concentrations [144]. A cohort study of 1489 middle-aged and older Chinese adults further showed that higher PFAS exposure—particularly to PFOA, PFNA, PFDA, and PFUnDA—was associated with reduced LTL. This effect was amplified by genetic susceptibility and dietary factors. Specifically, telomere shortening was more pronounced in individuals with a low healthy diet score and a high polygenic risk score for telomere attrition, based on 16 single-nucleotide polymorphisms identified in genome-wide analyses [145].

PFAS can cross the maternal–fetal barrier, and prenatal exposure has been shown to influence telomere length, potentially affecting susceptibility to age-related diseases later in life. These effects appear to be sex-specific and may be mediated by oxidative stress-related mechanisms. In a cohort of 581 newborns, higher PFOS and PFDA levels were associated with shorter LTL in female but not male infants, alongside increased ROS levels [146]. A similar study in a Chinese cohort confirmed inverse associations between maternal PFAS exposure and newborn telomere length and identified the spring birth season as a potential modifier of PFAS effects [147].

Mitochondrial DNA (mtDNA) is a key component of the cellular genome, organized as a double-stranded circular molecule encoding genes essential for mitochondrial function, particularly oxidative phosphorylation and energy metabolism. Unlike nuclear DNA, mtDNA lacks protective histones and has limited repair capacity, making it more vulnerable to oxidative damage. Consequently, mtDNAcn is considered a sensitive indicator of mitochondrial dysfunction and oxidative stress, processes implicated in atherosclerosis and, ultimately, in the development of IHD [148].

The overall influence of PFAS exposure on mtDNAcn appears to be heterogeneous and dependent on the specific congeners considered. In the study by Vriens et al., mtDNA content was higher in individuals with elevated PFOS serum concentrations, whereas exposure to PFHxS was associated with reduced mtDNAcn [144]. Similarly, a cross-sectional study conducted in China involving 453 participants reported positive associations between levels of perfluoro-3,5,7,9-butaoxadecanoic acid and perfluoro-3,5,7,9,11-pentaoxadodecanoic acid and mtDNAcn. Changes in mtDNAcn may reflect different biological responses: increases are often interpreted as compensatory mitochondrial biogenesis in response to stress, whereas decreases are generally associated with impaired mitochondrial replication or increased mitochondrial damage [115].

Consistent with observations for telomere length, mtDNAcn alterations have also been reported in relation to prenatal PFAS exposure. In a cohort of 572 mother–newborn pairs, maternal exposure to PFOS was inversely associated with mtDNAcn, while non-linear associations were observed for 2-(N-methyl-perfluorooctane sulfonamido)acetic acid, PFDA, and PFNA. A sex-specific pattern also emerged. Reductions in mtDNAcn were more pronounced in female newborns exposed to PFOS, whereas decreases were observed in male offspring in association with maternal PFHxS exposure. In the same study, folate status was identified as a potential modifier of PFAS-related mitochondrial effects [149]. Consistently, reduced mtDNAcn was also reported in a panel study including 284 children, where exposure to PFAS mixtures was associated with significant decreases in mtDNAcn, with PFOA identified as the main contributor [150].

As previously discussed, PFAS exposure has been associated with dyslipidemia, oxidative stress, and vascular dysfunction. However, not all exposed individuals develop cardiovascular disease, suggesting that genetic susceptibility may play a role in modulating risk. Gene–environment interactions may therefore contribute to inter-individual variability in disease onset and progression.

Single-nucleotide polymorphisms represent common genetic variations that can influence gene expression or function and modulate susceptibility to environmentally induced diseases, including PFAS-related cardiovascular effects [119]. In a study by Kobayashi et al. involving 504 pregnant women, variants in PPARD—a nuclear receptor regulating fatty acid oxidation and lipid homeostasis—were shown to modify the effects of PFOS exposure on lipid levels [151]. Notably, the rs1053049 TT genotype, which is associated with elevated LDL cholesterol and altered triglyceride and fatty acid profiles, may increase susceptibility to PFAS-related cardiometabolic disturbances that contribute to IHD development [151]. Similarly, in a cohort of 665 Faroese adults followed from birth, Valvi et al. identified several genetic variants, including ABCA1 rs3890182, FTO rs9939609 and rs3751812, PPARG rs170036314, and SLC12A3 rs2289116, that modify susceptibility to PFOS- and, to a lesser extent, PFOA-induced reductions in insulin sensitivity. These genes regulate lipid transport (ABCA1), energy balance and obesity (FTO, PPARG), and blood pressure (SLC12A3) [152]. Individuals carrying the corresponding risk alleles may be more susceptible to PFAS-related metabolic alterations linked to IHD [152].

## 6. Promising New Approaches to PFAS Research and Related Cardiovascular Risk Assessment

Digital Health represents the actual frontier of technology research in the field of healthcare and well-being [153], and in this framework, artificial intelligence (AI) presents a novel manner to answer common questions in the healthcare universe. More specifically, in the sector of environmental pollutants, it can be leveraged to improve understanding of PFAS-related cardiovascular toxicity and to enhance exposure assessment, risk prediction, and disease monitoring, particularly where early detection of exposure effects and related subclinical dysfunction becomes critical [154].

### 6.1. Potentialities of Artificial Intelligence for PFAS Exposure Assessment and Environmental Modeling

In the framework of PFAS-related research, it is of utmost importance to ensure proper classification of exposure due to the lack of completeness of historical data and, consequently, the heterogeneity of exposure pathways [155]. To cope with this, the integration of multiple heterogeneous data sources by means of AI can improve the whole exposure framework reconstruction [156], ultimately leading to the development of models that accurately estimate individual exposure trajectories to PFAS and overtake typical methodological limitations related to single biomarker measurements.

In addition, the increasing availability of high-dimensional biological data, such as epigenomic, transcriptomic, proteomic, metabolomic, and microbiome data, provides opportunities to identify complex associations between PFAS exposure and molecular pathways significantly concerned with cardiovascular disease [157,158].

AI, and machine learning (ML) in particular, as has been demonstrated for other conditions in the cardiovascular field [159], as well as in cancer research [160] and beyond, offers the possibility of detecting signatures of PFAS-induced endothelial dysfunction, mitochondrial impairment, lipid perturbation, and pro-inflammatory phenotypes. It also enables the integration of genomic susceptibility factors, with PFAS exposure data, which are potentially useful for the identification of subgroups at higher risk. All in all, AI modeling is foreseen to be capable of playing a role in clarifying the causal pathways and mediating factors related to the cardiovascular conditions associated with such exposures. For example, neural networks and ensemble learning techniques might be used to embed PFAS-related biomarkers along with traditional risk factors (lipids, blood pressure, glucose metabolism), lifestyle variables, medication history, and sociodemographic characteristics, to support early identification of individuals with subclinical endothelial dysfunction, increased arterial stiffness, or, more generally, enhanced vascular aging [161,162]. Longitudinal modeling fostered by AI could help clarify the dose–response patterns and highlight windows of susceptibility [163], particularly during critical life phases such as the prenatal or perinatal periods, adolescence or older age.

In practice, a study by Takeda and colleagues [164] attempted to build a dataset as input for ML models by testing exposure concentration and the components of nine straight-chain PFAS. This was ultimately aimed at assessing the mixed toxicity of perfluoroalkyl substances. To this end, a support vector machine was used to assess binary categorization between the cohort subjected to PFAS and controls. This analysis of the liver metabolome ultimately showed typical concentration-independent alterations upon PFAS exposure, including annotation of substances such as glutathione and 5-aminovaleric acid.

Another key contribution was provided by Yuan et al. [165], who combined molecular dynamics simulations with experimental data and used three ML algorithms, namely genetic function approximation, multiple linear regression, and neural network analysis, to establish a ΔG_water–interface QSAR model for predicting the removal effect of nanobubbles for PFAS in water. The results revealed an accuracy above 0.9 for the best-performing model (neural networks), further showing the promise of this approach in the related field of interest. From the actual perspective, one of the most challenging issues for the full-scale integration of such methods into practice is data quality, which is widely recognized as one of the major issues for AI applications in general terms and in this field in particular. However, with the rise in standardized protocols, this limitation would be probably overtaken in the near future, leading to enhanced data quality and ultimately benefitting the overall accuracy of AI-based models in the specific domain.

### 6.2. Future Perspectives for Consumer Technologies and Digital Health in PFAS-Related Effects Monitoring

Wearable tools are, like AI, solutions whose relevance in both the market and research is continuously growing, opening completely unprecedented opportunities for collecting continuous physiological data related to the health and well-being of an individual, particularly cardiovascular health in this case. Currently, modern biosensors and smartwatches have the capability of monitoring physiological parameters such as heart rate variability, blood pressure, oxygen saturation, sleep quality, physical activity and, indirectly, checking arrhythmia burden. These parameters are sensitive features to early signs of autonomic imbalance, endothelial dysfunction, and systemic inflammation, which may in turn be potentially influenced by PFAS exposure [166,167]. In addition, several IoT solutions have been explored for the detection of PFAS (and other EDCs) in sweat samples from human beings, sometimes with satisfying results (see [168] for a recent outline). A totally different perspective was explored by Niu and colleagues [169], who developed a personal wearable air sampler using sorbent-impregnated polyurethane foam, a famous compound thanks to its capability to capture PFAS, and compared it with silicone control wristbands. The device showed good performance in detecting both ionic and neutral PFAS, paving the way for a new avenue into personal devices for PFAS detection and quantification [169].

All things considered, the convergence of AI, multi-omics and consumer technologies leads to the implementation of precision medicine—and, in this case, precision cardiology—approaches that also turn out to be environmentally informed. Although some issues and challenges remain, such as data privacy, standardization, explainability and equitable access, those approaches, when merged, have the potential to enable early detection of PFAS-related cardiovascular impairments, ultimately leading to targeted prevention strategies.

## 7. Conclusions

Despite global regulatory efforts to restrict the production of legacy PFAS and limit their dispersion and accumulation across environmental media, PFAS exposure remains a worldwide concern due to their environmental persistence, long biological half-life in humans, and the partly unknown spectrum of their toxicological effects. Their role in the occurrence of IHD has yet to be fully established, with studies conducted in the general population reporting positive, null, or even inverse associations. The variety of congeners assessed and the variability in exposure profiles across study populations have likely contributed to these conflicting results. Notably, the significant positive associations described in the literature largely involve the most widespread congeners—PFOS, PFDA, and PFNA—although these same compounds have also shown an apparent protective effect against IHD in some analyses, further contributing to the inconsistency of current evidence. Moreover, studies conducted in occupational settings have not reported any association between PFAS exposure and IHD mortality, while evidence on disease incidence remains sparse and characterized by substantial uncertainty. By contrast, more consistent findings have emerged from research evaluating the relationship between PFAS and markers of atherosclerosis and vascular dysfunction, particularly PFOS and PFNA. Nonetheless, these indications require confirmation through large multicenter prospective studies that include repeated exposure measurements and standardized protocols for PFAS congener selection, as well as careful adjustment for key confounders. Particular attention should be given to potential effect modification by age, sex, and comorbidities to improve the interpretability and generalizability of findings. All this can be done with large-scale investigations, eventually supported by novel wearable tools for PFAS detection on human biofluids and biosignals, merged with AI-based approaches for data analysis and interpretation, possibly enabling the collection of large datasets with good data quality, which represent an essential requirement for this approach.

Current evidence suggests that PFAS may contribute to cardiovascular risk through a combination of metabolic, inflammatory, structural, and oxidative stress-related alterations, along with more subtle molecular alterations involving epigenetic regulation and genetic integrity. Changes in epigenetic signature, telomere length and mtDNAcn point to possible underlying biological effects; however, their direct causal role in the development of IHD remains insufficiently established. In addition, inter-individual variability linked to genetic background highlights the importance of gene–environment interactions in shaping susceptibility to PFAS-related cardiovascular damage. From a translational perspective, these sources of biological heterogeneity suggest that PFAS exposure may also contribute to variability in cardiometabolic traits commonly targeted by pharmacological treatment. Although direct clinical evidence is limited, PFAS-related disturbances in lipid metabolism, inflammation, and receptor-mediated signaling pathways may influence therapeutic response, identifying a relevant area for future clinically oriented research. Importantly, although the epidemiological evidence remains partly inconsistent, the convergence of multiple mechanistic pathways strengthens the biological plausibility of the association. In particular, the combined evidence supporting oxidative stress, lipid metabolism disruption, and epigenetic alterations suggests that these mechanisms may act synergistically rather than independently in mediating disease risk. This mechanistic concordance provides an important framework for interpreting heterogeneous epidemiological findings, which may reflect differences in study design, exposure assessment, population susceptibility, or follow-up duration. Therefore, despite current uncertainties, the consistency of these biologically plausible pathways supports the need for continued experimental and longitudinal research aimed at clarifying causality and identifying vulnerable populations.

Taken together, these observations underscore the need for integrated, multidisciplinary research to clarify causal mechanisms, validate reliable biomarkers of effect, and identify population groups at increased risk—ultimately informing targeted prevention and public health strategies. Future studies should also address the combined effects of PFAS mixtures on both intermediate and clinical cardiovascular outcomes. This effort should be supported by mechanistic investigations in experimental models and by leveraging emerging opportunities offered by artificial intelligence and advanced analytical tools to better elucidate biological pathways and strengthen causal inference.

## Data Availability

No new data were created or analyzed in this study. Data sharing is not applicable to this article.

## Abbreviations

The following abbreviations are used in this manuscript:

ACS	Acute coronary syndrome
APFO	Ammonium perfluorooctanoate
Apo	Apolipoprotein
BMI	Body mass index
EDC	Endocrine-disrupting chemical
CHD	Coronary heart disease
CIMP	Carotid intima–media thickness
CRP	C-reactive protein
CVD	Cardiovascular disease
HDL	High-density lipoprotein
HWE	Healthy worker effect
IHD	Ischemic heart disease
IMT	Intima–media thickness
LDL	Low-density lipoprotein
LTL	Leukocyte telomere length
MI	Myocardial infarction
MtDNAcn	Mitochondrial DNA copy number
OR	Odds ratio
PFAS	Per- and polyfluoroalkyl substances
PFBS	Perfluorobutane sulfonic acid
PFDA	Perfluorodecanoic acid
PFHpA	Perfluoroheptanoic acid
PFHxA	Perfluorohexanoic acid
PFHxS	Perfluorohexane sulfonate
PFNA	Perfluorononanoic acid
PFOA	Perfluorooctanoic acid
PFDoA	Perfluorododecanoic acid
PFOS	Perfluorooctane sulfonic acid
PFOSA	Perfluorooctane sulfonamide
PFUnDA	Perfluoroundecanoic acid
POP	Persistent organic pollutant
SMR	Standardized mortality ratio
SVHC	Substance of very high concern
TWI	Tolerable weekly intake
